# Survival of maxillary and mandibular bonded retainers 10 to 15 years after orthodontic treatment: a retrospective observational study

**DOI:** 10.1186/s40510-019-0279-8

**Published:** 2019-07-22

**Authors:** Katharina E. Kocher, Meret C. Gebistorf, Nikolaos Pandis, Piotr S. Fudalej, Christos Katsaros

**Affiliations:** 1 Medical Faculty, School of Dental Medicine, Department of Orthodontics and Dentofacial Orthopedics, University of Bern, Freiburgstrasse 9, 3010 Bern, Switzerland; 2 Department of Orthodontics, Institute of Dentistry and Oral Sciences, Palacky University Olomouc, Palackeho 12, 771 00 Olomouc, Czech Republic; 3 Medical Faculty, Department of Orthodontics, Jagiellonian University, Montelupich Street 4, 30-155 Kraków, Poland

**Keywords:** Retention, Long-term retention, Fixed retainers, Retainer failure, Bond failure, Adverse effects

## Abstract

**Background:**

The long-term evidence regarding failures of fixed retainers is limited and the aim of this cohort study was to assess the long-term risk of failure of one type of maxillary and two types of mandibular fixed lingual retainers.

**Trial design:**

Retrospective cohort study.

**Methods:**

Eighty-eight patients in retention 10–15 years after orthodontic treatment were included. The type of failure; number of failures per tooth, per patient, and retainer; and adverse effects were assessed by (1) a questionnaire, (2) clinical examination, and (3) screening patients’ clinical charts. Descriptive statistics were calculated and a Cox regression was used to assess possible predictors for mandibular retainer survival.

**Results and conclusions:**

In the mandible, 47 (53.4%) .016″ × .022″ braided stainless steel retainers (SS) were bonded to all six anterior teeth, and 41 (46.6%) .027″ β-titanium (TMA) retainers were bonded to the canines only. From the SS retainers 40.4% and of the TMA retainers 61% had no failures during the whole observation period. SS failures per retainer were 2.17 (3.15) vs. 0.66 (1.03) for TMA. The type of retainer was the only significant predictor for failure. In the maxilla, 82 (93.2%) .016″ × .022″ braided SS retainers were bonded to all four incisors and six retainers (6.8%) to all six anterior teeth. The latter group was not further analyzed due to the small sample size. From the retainers bonded to all four incisors, 74.4% had no failure during the whole observation period. SS average number of failures per retainer bonded to the four incisors was 1.14 (SD 2.93). Overall, detachments were the most frequent type of first failure followed by composite damage. From the original mandibular retainers 98.9% and of the original maxillary retainers 97.6% were still in situ 10–15 years after debonding. No adverse torque changes were observed.

**Limitations:**

Potential effects of selection bias, information bias, and attrition bias as well as possible confounding factors cannot be fully excluded in this study.

## Introduction

Today, most patients treated orthodontically expect an attractive smile for life. In order to meet the patients’ expectations, lifelong retention is frequently recommended [1]. However, extended wear of retainers is not free of failures and adverse effects. Depending on the type of retainer—removable or fixed—failures can range from breakage of the removable appliance to fracture of the wire bonded to the teeth. Moreover, in patients with long-term fixed retention, occlusal changes have been observed such as unexpected torque changes between adjacent teeth or opposite inclinations of contralateral mandibular canines [2]. In extreme situations, destruction of the buccal alveolar bone and development of gingival recession have been observed [3–6].

Failures of bonded retainers range from relatively easy to fix detachments of the wire from an individual tooth to detachment of the wire from several/all teeth resulting in retainer loss. Commonly, failures occur at the enamel-adhesive junction [7] and have been associated with moisture control, enamel contamination during the bonding procedures, and/or insufficient cleaning of the enamel prior to bonding. Failures at the adhesive-wire interface are less frequent. Regardless of the location and severity of the failure, repair is required because failures can promote plaque accumulation, discoloration, caries or undesirable tooth movement. Sometimes, the tooth detached from the retainer can move causing esthetic problems and the need for retreatment. Thus, periodic check-ups of retainers during the retention period are necessary.

Despite the large number of available retention protocols [1, 8, 9], there is a paucity of high-quality evidence in terms of the optimal fixed retention regimen [10–12]. Retention is a long-term process and prospective clinical trials are costly and likely to suffer from significant losses to follow-up which can jeopardize the validity of the results. This is a fairly common problem in studies dealing with retention procedures. Considering the large number of different retention regimens used by orthodontists, it is difficult to imagine that all of them will be tested through RCT. Retrospective studies, with long observation periods using different retention regimens, can be useful in terms of assessing fixed retention protocols and can provide insight into more targeted randomized trials.

To our knowledge, no retainer made from β-titanium (TMA) wire bonded only to mandibular canines has ever been assessed long-term. The aim of this study is to assess the survival and risk of failure 10 and 15 years after treatment of a maxillary retainer (.016″ × .022″ braided stainless steel (SS) wire bonded to all four anterior teeth) and two different mandibular retainers (I) .016″ × .022″ braided SS wire bonded to all six mandibular anterior teeth (incisors and canines) and (II) .027″ round TMA wire bonded to mandibular canines only.

## Materials and methods

For this retrospective cohort study, the longitudinal sample reported by Gebistorf et al. 2018 [13] was used with slightly modified inclusion and exclusion criteria. No prior sample size calculation was performed; however, all eligible patients were considered. The study was approved by the Ethic Committee of Bern, Northwest- and Central Switzerland (EKNZ 2015-349, HVF, Kat A) and every patient signed an informed consent before inclusion in the study. The STROBE guidelines for reporting of observational studies were followed for the structure of the present article [14].

### Participants

The sample was selected of patients from a private orthodontic practice in Switzerland. In this practice, it was routine to keep pre-treatment (i.e., initial, *T*_1_) and post-treatment records (i.e., final, *T*_2_) for at least 10 years after the last retainer check-up visit. The last visit was usually performed between 1 and 4 years post-treatment before patients were referred to their private dentist [13]. A two-phase treatment for growth modification with a removable appliance, extractions, interproximal enamel reduction or surgery was applied if necessary according to the orthodontic treatment protocol. No circumferential supracrestal fiberotomy was performed.

Three hundred ninety-four consecutive patients with their post-treatment (*T*_2_) records taken between the years 2001 and 2006 were contacted for a recall appointment if they met the following inclusion criteria: (a) treated with fixed appliances; (b) treated by the same orthodontist; (c) maxillary and mandibular retainers bonded immediately after completion of active orthodontic treatment; and (d) non-syndromic patients. No age restriction was applied during the sample selection.

**Fig. 1:**
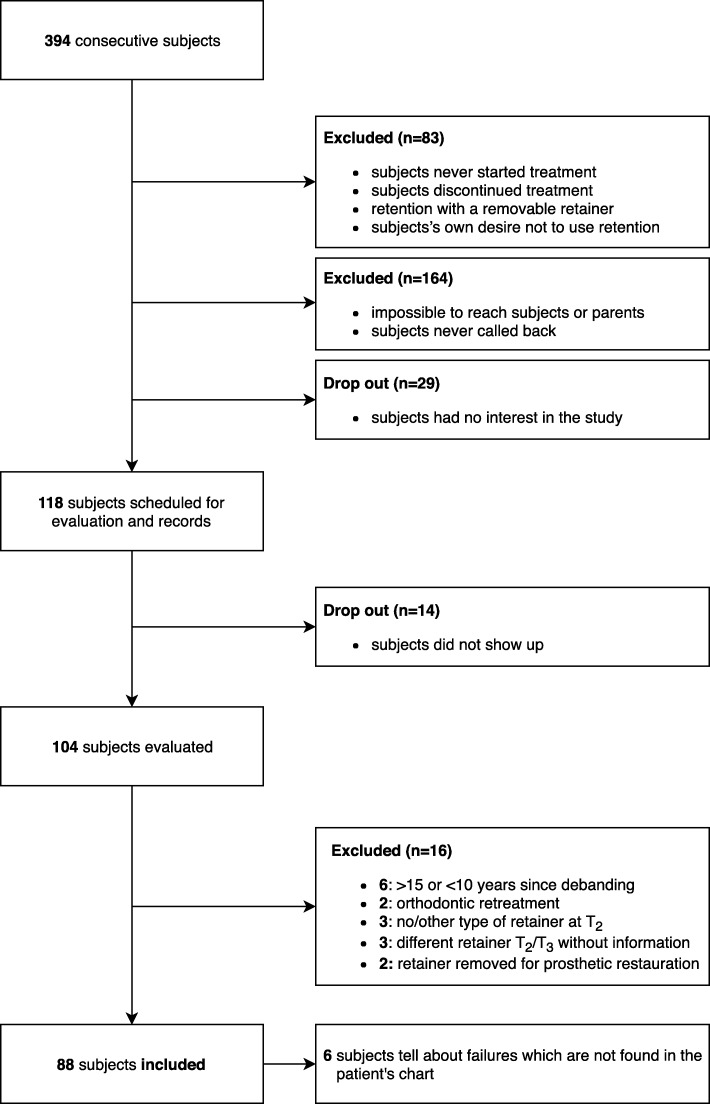
Flow chart of the study participants

The flow chart (Fig. 1) shows in detail the procedure of patient selection. Eighty-three patients never received any fixed retainer and they were excluded in this study (never started/discontinued treatment, no/removable retention device). Another 164 patients could not be contacted (unavailable contact information/patients never called back). From the remaining 147 patients, 118 patients agreed to participate, but 14 of them did not come to the scheduled appointment. One hundred four patients were finally evaluated. The recall appointment involved (I) a clinical examination, (II) taking pictures, and (III) cast impressions [13]. After the recall appointment, the additional exclusion criteria were applied for the present study: (a) orthodontic retreatment, (b) post-treatment appointment (i.e., retention time, *T*_3_) less than 10 or more than 15 years ago, (c) retention phase with no/other mandibular lingual retainer than (I) .016″ × .022″ braided SS bonded to all six mandibular anterior teeth or (II) .027″ round TMA bonded canine to canine and maxillary retainer .016″ x.022″ braided SS bonded to all four or six anterior maxillary teeth, (d) different/modified mandibular or maxillary retainer in situ at *T*_2_ and *T*_3_ without information, and (e) mandibular or maxillary retainer removed for prosthetic restorations. Eighty-eight patients were finally included for the present study.

### Retention protocol

**Fig. 2:**
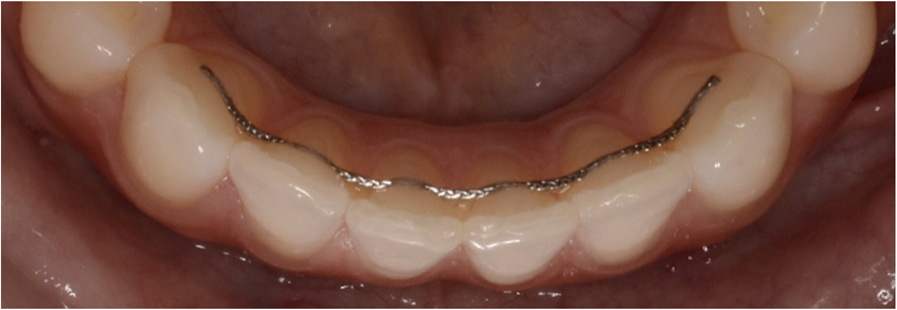
.016″ × .022″ eight-strand braided SS wire (D-Rect., ORMCO) bonded to all 6 lower anterior teeth at *T*_3_

**Fig. 3:**
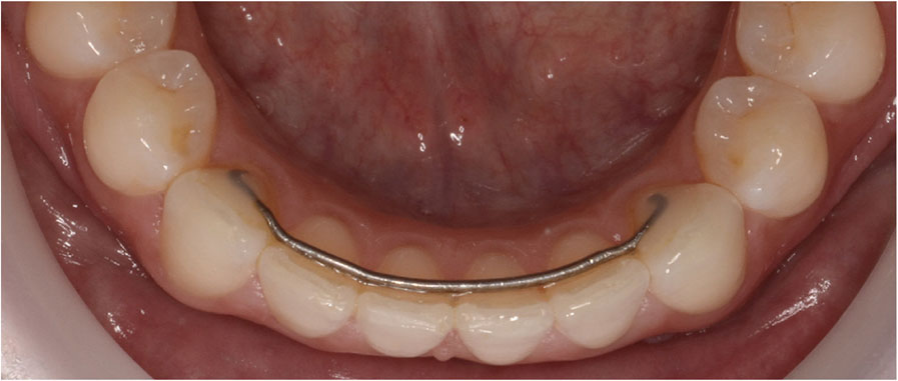
.027″ round TMA wire (ORMCO) bonded to lower canines only at *T*_3_

Two types of mandibular retainers were used in participants: (I) .016″ × .022″ eight-strand braided SS wire (D-Rect., ORMCO) bonded to all six lower anterior teeth (incisors and canines) (Fig. 2) and (II) .027″ round TMA wire bonded to canines only (ORMCO) (Fig. 3).

The TMA wires were sandblasted at the bonding sites. The decision for the type of retainer was made by the orthodontist according to the oral hygiene status and the initial amount of crowding. In patients with good hygiene and/or significant initial crowding, a retainer bonded to all six lower anterior teeth was chosen. In patients with poor oral hygiene and/or little initial crowding, a TMA retainer bonded only to the canines was administered. The initial amount of crowding was considered more important than the current level of oral hygiene for the choice of the type of retainer; thus patients with large initial crowding and poor oral hygiene were retained with .016″ × .022″ braided SS wires bonded to all six anterior teeth.

**Fig. 4:**
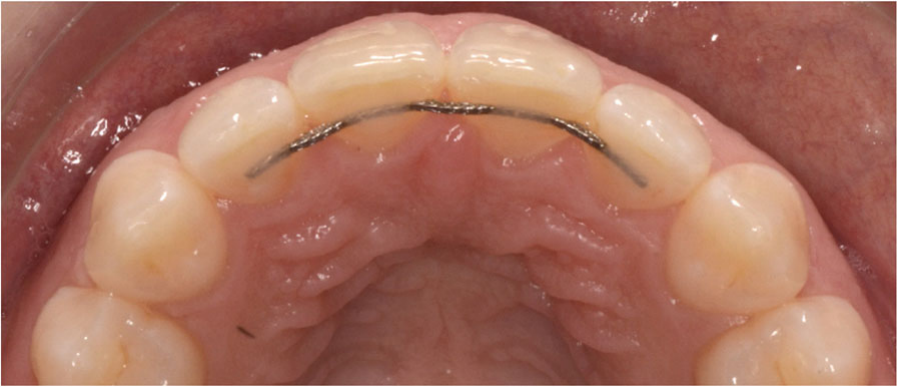
016″ × .022″ eight-strand braided SS wire (D-Rect., ORMCO) bonded to all four maxillary incisors at *T*_3_

The standard retainer in the maxilla was .016″ × .022″eight-strand braided SS wire (D-Rect., ORMCO) bonded to all four incisors (Fig. 4). For six patients, the wire was extended to the canines because they were very severely displaced before treatment (*T*_1_).

All retainers were bonded following a standardized procedure by the same orthodontist. The tooth surfaces were cleaned with a low-speed hand piece using a rubber cap with non-fluoridated pumice and sandblasted. Then the enamel was etched with 37% phosphoric acid gel for 30 s, washed and air dried. The bonding (Ortho Solo. ORMCO) was applied and light cured for 5 s per tooth. The retainers were manually placed in the correct position on the teeth, stabilized with a high viscous composite (Charism. KULZER, Mitsui Chemicals Group), and then covered with a thin layer of flowable composite (Flow Tain. ORTHOBY).

### Data collection

The primary outcome for the present study was retainer failure. The type of failure and the time point failures occurred were registered based on: (a) a questionnaire answered by the participants at the recall appointment 10–15 years after debonding (*T*_3_), (b) clinical examination by two calibrated examiners at a recall appointment 10–15 years after debonding (*T*_3_), and (c) patients’ clinical charts, which were screened for notes about failures by one examiner (KK). Eight categories of different types of failures were defined before the clinical examination:

- 0: intact bonding
- 1: full retainer out and rebonded
- 2: fracture of the wire
- 3: detachment
  (at the wire-composite interface or adhesive-enamel interface—rebond of the tooth to the retainer necessary)
- 4: composite damage
  (chipping within the composite—retainer still attached to the tooth but some composite necessary to recover the wire)
- 5: retainer replaced by new retainer
- 6: no retainer in situ at *T*_3_
- 7: multiple failures at the same time

The time point of each failure was registered in months (m) after debonding. Whenever patients mentioned failures which were not found in their corresponding clinical charts, the failures were counted as they were probably restored at the private dentist’s office, but the time point was omitted since it was not available. Data retrieval from the three different sources was accomplished within 4 weeks. Demographic data such as patients’ gender, age, and treatment duration were obtained from the clinical charts (Table 1). If there was suspicion of an adverse effect (i.e., torque/movement of teeth with intact bonding sites), the casts were visually inspected and judged by a second experienced clinician (CK). Other adverse effects (decalcifications/periodontal parameters) were not considered in the present study. The effectiveness of the different wires to maintain alignment and prevent relapse will be assessed in a separate paper.

**Table 1:** Baseline characteristics of the two mandibular and the two maxillary retainer groups

		Mandible			Maxilla		
		.016″ × .022″ braided SS 6 incisors (*N* = 47) mean (SD) or (%)	.027″ TMA (*N* = 41) mean (SD) or (%)	total mean (SD) or (%)	.016″ × .022″ braided SS 4 incisors (*N* = 82) mean (SD) or (%)	.016″ × .022″ braided SS 6 incisors (*N* = 6) mean (SD) or (%)	total mean (SD) or (%)
Age at *T*_3_ (year)		27.8 (2.9)	27.9 (1.8)	27.8 (2.4)	27.7 (2.3)	29.9 (2.6)	27.8 (2.4)
Gender	Male	12 (25.5%)	13 (31.7%)	25 (28.4%)	24 (29.3%)	1 (16.7%)	25 (28.4%)
	Female	35 (74.5%)	28 (68.3%)	63 (71.6%)	58 (70.7%)	5 (83.3%)	63 (71.6%)
Duration (year)	Treatment (*T*_1_–*T*_2_)	3.1 (1.5)	2.9 (1.1)	3.0 (1.3)	2.9 (1.2)	4.2 (1.9)	3.0 (1.3)
	Retention (*T*_2_–*T*_3_)	12.6 (1.2)	13.3 (1.1)	12.9 (1.2)	12.9 (1.2)	12.4 (1.4)	12.9 (1.2)

### Statistical analysis

Descriptive statistics were calculated including baseline characteristics, type of first failure, distribution and number of failures per tooth type, and number of failures per patient and retainer. The maxillary and mandibular retainers were analyzed separately. Possible predictors for mandibular retainer failure were assessed using Cox regression excluding six patients with unknown time to first failure. Significance level for all statistical tests was predetermined at 0.05. All analyses were performed using Stata 15 statistical software (Statacorp, College Station, TX, USA).

## Results

Table 1 summarizes the patient demographic characteristics. The sample consisted of 63 (71.6%) female and 25 (28.4%) male patients.

The mean follow-up period for the mandibular .016″ × .022″ braided SS retainers was slightly shorter than that of the .027″ round TMA retainers (12.6 vs. 13.3 years). Mean treatment duration, age at *T*_3_, as well as the gender distribution were similar in the two mandibular retention groups.

In the maxilla, most patients (93.2%) had a .016″ × .022″ braided SS wire bonded to all four maxillary incisors. In 6.8% of the patients, the retainer was bonded to all maxillary incisors and canines. Failures of the maxillary retainers bonded to six anterior teeth were not further analyzed due to the small number of patients in this group.

The distribution of the various types of first failure at the patient level is shown in Table 2 for the mandibular and in Table 3 for the maxillary retainers.

**Table 2:** Type of first failure for the mandible

	.016″ × .022″ braided SS (*N* = 47)			.027″ TMA (*N* = 41)			Total for both retainers (*N* = 88)
	Male (*N* = 12)	Female (*N* = 35)	Total (N = 47)	Male (*N* = 13)	Female (*N* = 28)	Total (N = 41)	
0: intact bonding	5	14	19 (40.4%)	5	20	25 (61%)	44 (50%)
1: full retainer out and rebonded	1	0	1	3	2	5	6 (6.8%)
2: fracture	0	0	0	0	0	0	0 (0%)
3: detachment	0	10	10	4	3	7	17 (19.3%)
4: composite damage	5	11	16	1	3	4	20 (22.7%)
5: retainer replaced by a new retainer	0	0	0	0	0	0	0 (0%)
6: no retainer in situ at T_3_	1	0	1	0	0	0	1 (1.1%)
7: multiple types of failures at the same time	0	0	0	0	0	0	0 (0%)
Total failures per type of retainer	–	–	28 (59.6%)	–	–	16 (39%)	–
Total failures for both retainers							44 (50%)

**Table 3:** Type of the first failure for the maxilla

	.016″ × .022″ braided SS four incisors (*N* = 82)		
	Male (*N* = 24)	Female (*N* = 58)	Total (*N* = 82)
0: intact bonding	13	48	61 (74.4%)
1: full retainer out and rebonded	0	0	0 (0%)
2: fracture	0	0	0 (0%)
3: detachment	3	3	6 (7.3%)
4: composite damage	6	5	11 (13.4%)
5: retainer replaced by a new retainer	0	0	0 (0%)
6: no retainer in situ at T_3_	1	1	2 (2.4%)
7: multiple types of failures at the same time	1	1	2 (2.4%)
Total failures per retainer			21 (25.6%)

No failure of any type was observed in the mandible for 19 (40.4%) patients fitted with the .016″ × .022″ braided SS retainers and 25 (61%) with the .027″ round TMA retainers. Table 4 shows the results from the Cox model for the mandibular retainers. The only significant predictor for survival was the type of retainer (hazard ratio 0.42, 95% CI 0.22, 0.81 for braided SS vs. TMA, *p* = 0.009).

**Table 4:** Cox regression for the effect of retainer type on mandibular lingual retainer survival adjusted for age at *T*_3_ and gender

Predictor		Hazard ratio	95% CI		*P* value
Type of retainer	.016″ × .022″ braided SS	Reference			
	.027″ TMA	0.42	0.22	0.81	0.009
Age at *T*_3_ (per unit = 1 year)		1.09	0.98	1.2	0.121
Gender	Male	Reference			
	Female	0.55	0.28	1.1	0.085

In the maxilla, 61 (74.4%) retainers never experienced any failure during the whole observation period. In both jaws, the most frequent first failure was composite damage (mandible 22.7%; maxilla 13.4%) followed by detachment (mandible 19.3%; maxilla 7.3%). Six events 6.8% of loss of the whole retainer with need for replacement in the mandible were observed. No retainer experienced any fracture. Serious first failures, other than composite damages and detachments, occurred in 7.9% in the mandible and 4.8% in the maxilla. The original retainer was still in situ 10–15 years after debonding for 87 patients (98.9%) in the mandible and for 80 patients (97.6%) in the maxilla.

The distribution of failures according to the tooth type is shown in the Tables 5, 6, and 7. In the mandible, on single tooth level, no failure was observed for 75.1% of the 282 bonded teeth with .016″ × .022″ braided SS wire and for 71.9% of the 82 bonded teeth with .027″ round TMA wire respectively. For the .016″ × .022″ braided SS wire, bonded sites on the canines showed on average a higher survival rate than bonded sites on incisors (85.1% vs. 70.3%). Comparing the bonded sites on the canines for the two wires, less failures were observed for the .016″ × .022″ braided SS wire than for the .027″ round TMA wire (85.1% survival vs. 71.9%). Only 7.2% of the bonded teeth with a .016″ × .022″ braided SS retainer and 4.9% of the teeth with a .027″ round TMA retainer failed two or more times.

**Table 5:** Number of failures according to tooth type in the mandible for the *.*016″ × .022″ braided SS retainers (47 patients, 282 total bonded sites)

Number of failures	43 (%)	42 (%)	41 (%)	31 (%)	32 (%)	33 (%)	Total (%)
0 (no failures)	38 (80.9%)	35 (74.5%)	34 (72.4%)	30 (63.9%)	33 (70.3%)	42 (89.4%)	212 (75.1%)
1	7 (14.9%)	6 (12.8%)	11 (23.4%)	11 (23.4%)	11 (23.4%)	4 (8.5%)	50 (17.7%)
2	1 (2.1%)	5 (10.6%)	1 (2.1%)	3 (6.4%)	3 (6.3%)	1 (2.1%)	14 (5.0%)
3	1 (2.1%)	1 (2.1%)	0 (0%)	1 (2.1%)	0 (0%)	0 (0%)	3 (1.1%)
4	0 (0%)	0 (0%)	1 (2.1%)	1 (2.1%)	0 (0%)	0 (0%)	2 (0.7%)
7	0 (0%)	0 (0%)	0 (0%)	1 (2.1%)	0 (0%)	0 (0%)	1 (0.4%)
Total number of failures (%)	9 (19.1%)	12 (25.5%)	13 (27.6%)	17 (36.1%)	14 (29.7%)	47 (10.6%)	70 (24.9%)

**Table 6:** Number of failures according to tooth type in the mandible for the *.*027″ round TMA retainers (41 patients, 82 total bonded sites)

Number of failures	43 (%)	33 (%)	Total (%)
0 (no failures)	31 (75.6%)	28 (68.3%)	59 (71.9%)
1	8 (19.5%)	11 (26.8%)	19 (23.2%)
2	2 (4.9%)	2 (4.9%)	4 (4.9%)
Total number of failures (%)	10 (24.4%)	13 (31.7%)	23 (28.1%)

**Table 7:** Number of failures according to tooth type in the maxilla for the .016″ × .022″ braided SS retainers bonded to 4 incisors (82 patients, 328 bonded sites)

Number of failures (%)	12 (%)	11 (%)	21 (%)	22 (%)	Total (%)
0 (no failures)	66 (80.5%)	72 (87.9%)	71 (86.6%)	74 (90.2%)	283 (86.3%)
1	14 (17.1%)	7 (8.5%)	8 (9.8%)	7 (8.5%)	36 (11.0%)
2	1 (1.2%)	2 (2.4%)	2 (2.4%)	0 (0%)	5 (1.5%)
3	0 (0%)	1 (1.2%)	1 (1.2%)	1 (1.2%)	3 (0.9%)
4	1 (1.2%)	0 (0%)	0 (0%)	0 (0%)	1 (0.3%)
Total number of failures (%)	16 (19.5%)	10 (12.1%)	11 (13.4%)	8 (9.8%)	45 (13.7%)

In the maxilla, 86.3% of the 328 bonded teeth with .016″ × .022″ braided SS wire on four incisors showed no failure. No specific pattern of the number of failures of bonded sites between central or lateral incisors could be observed. Two or more failures were observed by 2.7% of the bonded sites.

**Fig. 5:**
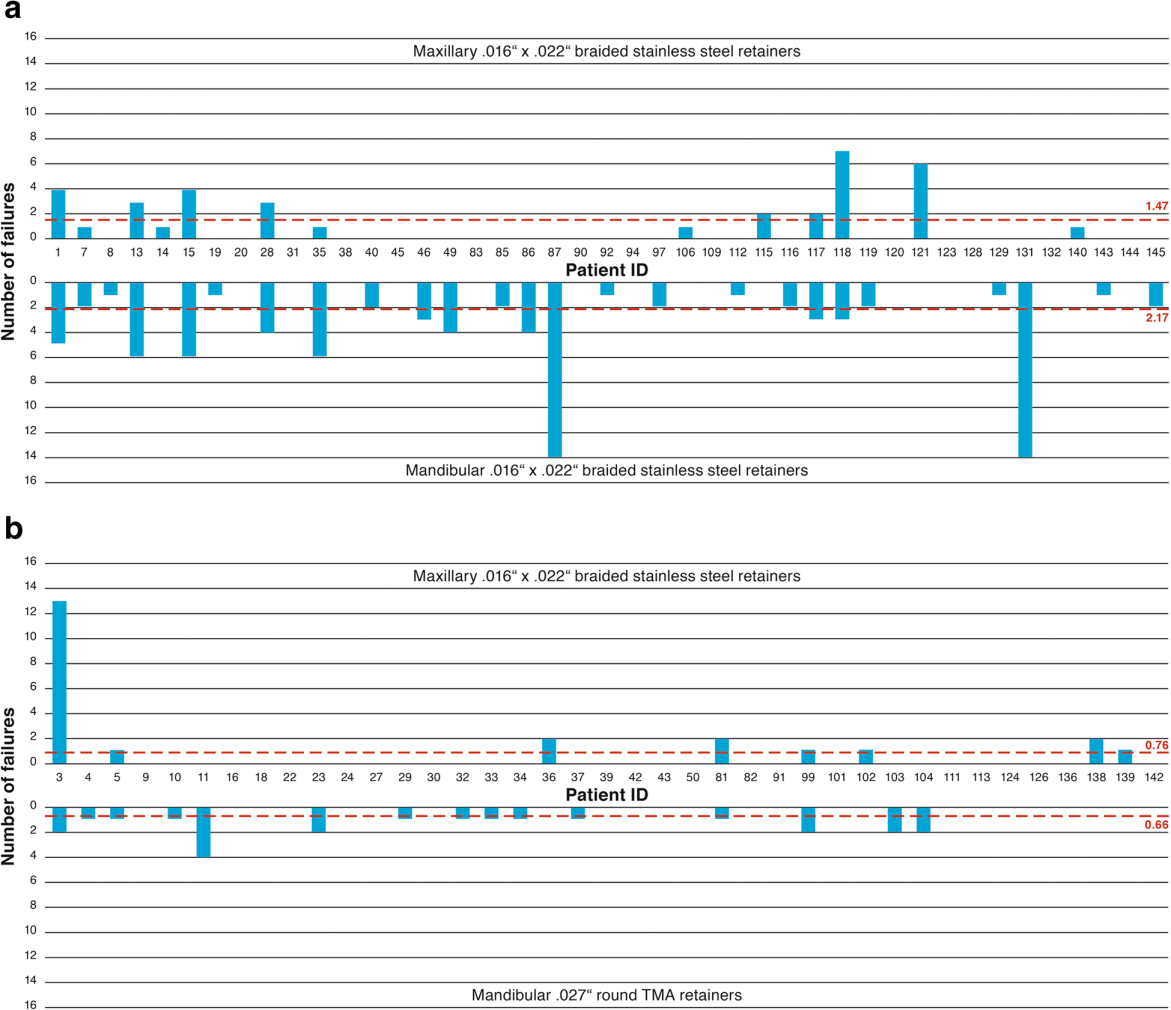
**a** Failures per patient for the group with mandibular .016″ × .022″ braided stainless steel retainers bonded to all six anterior teeth and maxillary .016″ × .022″ braided stainless steel retainers bonded to all four anterior teeth. **b** Failures per patient for the group with mandibular .027″ round TMA retainers bonded only to the canines and maxillary .016″ × .022″ braided stainless steel retainers bonded to all four anterior teeth

The average number of failures per patient according the jaw and the type of retainer (Table 8) was greater (2.17, SD = 3.15) for .016″ × .022″ braided SS retainers compared to .027″ round TMA retainers (0.66, SD = 1.03) in the mandible. Figure 5a demonstrates that multiple failures were limited to only a few patients for the .016″ × .022″ braided SS retainers (median 1.0). The distribution of the number of failures for the .027″ round TMA retainers is within a smaller range (median 0) (Table 8, Fig. 5b).

**Table 8:** Failures per patient in the mandible and maxilla according to the type of retainer

		Mean (SD)	Median	p25	p50	p75
Mandible	.016″ × .022″ braided SS (*N* = 47)	2.17 (3.15)	1.0	0	1	3
	.027″ TMA (*N* = 41)	0.66 (1.03)	0	0	0	1
	Total (*N* = 88)	1.47 (2.52)	0.5	0	0.5	2
Maxilla	.016″ × .022″ braided SS 4 incisors (*N* = 82)	1.14 (2.93)	0	0	0	1

p25 = lower quartile (25%); p75 = upper quartile (75%)

In the maxilla, we observed 1.14 failures per retainer per patients (Table 8). Figure 5b shows that comparable to the mandibular .016″ × .022″ braided SS retainers, only few patients contributed to multiple failures (median 0).

In respect of adverse effects, one lateral maxillary incisor retained with a .016″ × .022″ braided SS wire bonded to the four incisors showed a slight disto-buccal drift and one mandibular canine stabilized with a .016″ × .022″ braided steel drifted slightly toward buccal direction. No undesired changes of the root torque were observed in any patient.

## Discussion

Bonded retainers made of various materials and attached to different numbers of teeth are widely used to maintain alignment of the teeth after orthodontic treatment [1]. A long retainer survival is desirable for alignment maintenance and a less burdensome follow-up for the patient and the practice [15]. In this retrospective study, we analyzed survival of two different types of mandibular retainers and one maxillary retainer 10–15 years post-treatment.

To our knowledge, TMA retainers bonded only to canines have never been assessed long-term. This could be due to the fact that TMA retainers are rarely used by orthodontists as stainless steel retainers of various dimensions seem to be the retainers of choice [1]. We found that 10–15 years after debonding, TMA retainers were free of failures more often than the stainless steel ones bonded to all anterior teeth (61.0% vs. 40.4%, respectively). A recent systematic review [12] reported that the risk of failure for mandibular stainless steel retainers bonded from canine to canine and mandibular stainless steel retainers bonded to canines only is comparable (29% versus 26%, respectively). A discrepancy between our results and those of the systematic review can be explained by a significantly longer observation time in our investigation compared to the publications included in the review. The follow-up period of eight studies included in the quantitative analysis ranged from 6 to 36 months, while it was 120–180 months in our sample. One can assume that a longer observation corresponds with increased risk of failure. Renkema et al. observed after a 5-year observation period a higher risk of failure (31.7%) for .019″ three-strand, heat-treated twist wires bonded to all six mandibular anterior teeth than for .0215″ × .027″ SS rounded rectangular wires bonded to canines only (20.4%) [16, 17]. The risk of failure in our participants with TMA retainer is also very similar to that reported by Booth et al. [18]—62% patients with mandibular .025″ SS retainers bonded to the canines had no breakage over the minimum 20-year observation period.

Analysis of the failures per retainer showed that a .016″ × .022 SS retainer had on average 2.17 failures during follow-up, whereas a .027″ round TMA retainer had only 0.66 failures in a comparable period. This difference between the retainers should be interpreted carefully. First, a retainer bonded to all six anterior teeth has three times more bonded sites at risk for failure than a TMA retainer bonded to canines only (6 vs. 2). The difference of the risk of failure rate is very small if we take this into account. Second, only few patients contributed to multiple failures and these patients increased the mean value for the total number of failures. Only 7.2% of the .016″ × .022″ braided SS mandibular retainers and 4.9% of the .027″ round TMA mandibular retainers failed twice or more times. Our findings are in accordance with Scheibe and Ruf [19] who found that 4.5% patients had more than two detachments in a 3-year observation period.

At the tooth level and considering the above, the survival of the mandibular bonded sites is even slightly higher for the .016″ × .022 SS retainers than for the .027″ round TMA retainers (75.1% survival vs. 71.9%). We could see that for the .016″ × .022″ braided SS retainers, survival differed between incisors (70.3%) and canines (85.1%). Focusing only at the bonded sites of the canines, the difference between the two wires is further accentuated (85.1% survival for the SS retainers vs. 71.9% for the TMA retainers). Thus, the difference in survival of the two retainers overall (TMA 61.0% vs. SS 40.4%) is mainly due to the different wire design in terms of the number of bonding sites. This assumption is supported by our finding that the type of retainer is the only significant predictor for mandibular retainer survival. There was no evidence that gender or patient age at *T*_3_ are significant predictors for retainer survival.

In the maxilla, 25.6% of the .016′′ × .022 braided SS retainers (TMA retainers were not used for retention of maxillary teeth) bonded to four incisors failed at least once during observation. Unfortunately, no meta-analysis of failures of the maxillary bonded retainers was reported in the existing systematic reviews [10, 12]. As a result, our findings can be compared only with results of other individual studies. In general, significant heterogeneity exists in the literature with some studies reporting lower failure rates of maxillary bonded retainers [20–22] and others reporting higher failure rates [23–25] than in our study. The range of failure prevalence observed in other investigations was from 6.2% after 4.5 years with .0215″ gold-coated wires [20] to 48.2% after 6 years with three-stranded spiral wire [23]. According to Zachrisson [20], extension of the maxillary retainer to the canines seems to increase the risk for failures. Since only six patients in our sample had a maxillary retainer bonded to all six anterior teeth, we could not relate to the statement of Zachrisson.

We found that a maxillary retainer failed on average 1.14 times. Similar to the mandibular retainers, few patients had multiple failures. Two or more failures were observed only for 2.7% of the maxillary .016″ × .022″ braided SS retainers bonded to four anterior teeth. Therefore, the mean number of failures per retainer is somewhat inflated because it is artificially increased by those with several failures.

Composite damage was the most frequent type of failure in both jaws followed by detachment. Salehi et al. [26] described retainer loosening as the most frequent type of failure for 0.0175″ flexible spiral wires (96.4% in the mandible, 81.5% in the maxilla). Dietrich et al. [25] reported that 85.7% incidents were detachments; also, Tacken et al. [21] found that ten out of 13 failures in the maxilla and 100% of the failures in the mandible were detachments. Forde et al. [27] described that most of the maxillary failures tended to occur between the wire and composite, whereas mandibular failures were more common at the enamel-composite interface. Jin et al. [28] described debonding (without differentiating between composite damage and detachment) as the main reason for failure of .016 × .022″ SS wires bonded to the canines only. Both types of retainer failure—composite damage and detachment—are of minor clinical importance with regards to alignment stability if detected early but if overlooked, they can lead to relapse of tooth alignment. Therefore, regular recall appointments to assess the integrity of the retainers are important.

Complete detachments of the wire were not a common occurrences in our study and no fractures were found either in the mandible or in the maxilla. These types of failure usually require a new retainer with increased associated costs and chair-side time compared to single composite damages and detachments. Our findings agree with reports of other authors [17, 27, 29].

Ten to fifteen years after debonding, 98.9% of the original mandibular retainers and 97.6% of the original maxillary retainers were still in situ. These high survival values for the retainers as a whole unit show that a wire can continue to function well after adequate repair of a single failure on tooth level site.

In terms of side effects, no severe complications such as torque differences between adjacent teeth as described by Katsaros et al. [2] for the round flexible spiral retainers were found with neither type of retainers. Probably the .016″ × .022″ eight-stranded braided SS wire is more resistant to post-treatment activation in comparison to the round flexible wires. The slight buccal movement of one maxillary incisor and one mandibular canine at the end of the .016″ × .022″ braided SS wires could be possibly attributed to slight wire activation at the time of bonding.

A strength of our study is that all retainers were bonded by the same operator with > 10 years of experience in placing retainers [28] and the long follow-up period. The fact that this study was carried out in a single office makes the results however less generalizable.

We did not further assess any periodontal parameters for the present study. A recent systematic review [12] found however no significant differences regarding periodontal outcomes between mandibular stainless steel fixed retainers bonded to all anterior teeth or the canines only.

Apart from the failures, the effectiveness of a retainer to maintain alignment is crucial in the decision-making process of wire material and design selection. We will addressed this topic in a future separate paper.

## Limitations

The material for the study was obtained from a private practice in Switzerland. Our sample can be subject to self-selection bias because, for example, patients pleased with the treatment result might be more willing to participate in a follow-up examination.

A fairly high response rate 104/147 (70.7%) could have still biased our findings. It is impossible to determine the direction and size of the influence of the losses on our results, i.e., if participants dropped-out from the study had a higher, comparable, or lower failure rate than the ones who remained in the study. An effort was made to assess any failure by three different methods (questionnaire, clinical examination, screening clinical charts); however, we cannot rule out information bias as some failures may have been treated by a private dentist and were not reported by patients. Therefore, the true level of failures could have been underestimated. Furthermore, performance and / or detection bias cannot be precluded as no blinding was implemented during follow up and outcome recording.

The pre-treatment malocclusion could be a possible confounder.

The fact that this study was carried out in a single office and that the choice for the type of the mandibular retainer and the bonding procedures were always carried out by the same clinician makes the results less generalizable.

## Conclusions

Within the limitations of this retrospective study, we conclude that:

1. A higher percentage (61%) of the mandibular .027″ round TMA retainers bonded only to the canines survived without any failures 10–15 years after debonding in comparison with the mandibular .016″ × .022″ braided SS wires bonded to all six mandibular anterior teeth (40.4%).
2. In the maxilla, a high percentage (74.4%) of the 016″ × .022″ braided SS retainers bonded to all four incisors survived without any failures during the whole observation period.
3. For all wires used in this study, the most common failures were composite damage and detachments. Severe failures and multiple failures (≥ 2) were found to be rare.
4. Further, 98.9% of the original mandibular retainers and 97.6% of the original maxillary retainers were still in situ 10–15 years after debonding.

## Abbreviations

CI: Confidence interval
m: Month(s)
s: Seconds
SD: Standard deviation
SS: Stainless steel
TMA: Beta titanium
vs: Versus
